# Zoonotic Blood-Borne Pathogens in Non-Human Primates in the Neotropical Region: A Systematic Review

**DOI:** 10.3390/pathogens10081009

**Published:** 2021-08-10

**Authors:** Gabriel Carrillo-Bilbao, Sarah Martin-Solano, Claude Saegerman

**Affiliations:** 1 Research Unit of Epidemiology and Risk Analysis Applied to Veterinary Sciences (UREAR-ULiège), Fundamental and Applied Research for Animal and Health (FARAH) Center, Department of Infections and Parasitic Diseases, Faculty of Veterinary Medicine, University of Liège, 4000 Liège, Belgium; gabriel.carrillobilbao@doct.uliege.be; 2 Facultad de Filosofía y Letras y Ciencias de la Educación, Universidad Central del Ecuador, 170521 Quito, Ecuador; 3 Instituto de Investigación en Zoonosis (CIZ), Universidad Central del Ecuador, 170521 Quito, Ecuador; ssmartin@espe.edu.ec; 4 Grupo de Investigación en Sanidad Animal y Humana (GISAH), Carrera Ingeniería en Biotecnología, Departamento de Ciencias de la Vida y la Agricultura, Universidad de las Fuerzas Armadas—ESPE, 171103 Sangolquí, Ecuador

**Keywords:** Ecuador, non-human primates, *Alouatta*, blood-borne pathogen, protozoa, *Plasmodium*, *Trypanosoma*, yellow fever

## Abstract

**Background**: Understanding which non-human primates (NHPs) act as a wild reservoir for blood-borne pathogens will allow us to better understand the ecology of diseases and the role of NHPs in the emergence of human diseases in Ecuador, a small country in South America that lacks information on most of these pathogens. **Methods and principal findings**: A systematic review was carried out using PRISMA guidelines from 1927 until 2019 about blood-borne pathogens present in NHPs of the Neotropical region (i.e., South America and Middle America). **Results**: A total of 127 publications were found in several databases. We found in 25 genera (132 species) of NHPs a total of 56 blood-borne pathogens in 197 records where Protozoa has the highest number of records in neotropical NHPs (*n* = 128) compared to bacteria (*n* = 12) and viruses (*n* = 57). *Plasmodium brasilianum* and *Trypanosoma cruzi* are the most recorded protozoa in NHP. The neotropical primate genus with the highest number of blood-borne pathogens recorded is *Alouatta* sp. (*n* = 32). The use of non-invasive samples for neotropical NHPs remains poor in a group where several species are endangered or threatened. A combination of serological and molecular techniques is common when detecting blood-borne pathogens. Socioecological and ecological risk factors facilitate the transmission of these parasites. Finally, a large number of countries remain unsurveyed, such as Ecuador, which can be of public health importance. **Conclusions and significance**: NHPs are potential reservoirs of a large number of blood-borne pathogens. In Ecuador, research activities should be focused on bacteria and viruses, where there is a gap of information for neotropical NHPs, in order to implement surveillance programs with regular and effective monitoring protocols adapted to NHPs.

## 1. Introduction

Wild animals are the cause of almost 70% of all emerging diseases [1], and more than 60% of these diseases are zoonotic [2]. This is a public health concern and a conservation problem [3,4]. Non-human primates (NHPs) are infected not only by gastrointestinal parasites [5], but also by ectoparasites, hemoparasites, bacteria, viruses and some arthropods that affect the lungs. Until recently [6,7], just a few studies identified blood pathogens from fecal samples due to the presence of DNA (deoxyribonucleic acid) inhibitors in fecal samples. Thereafter, just a few studies have identified hemoparasites such as *Plasmodium* sp. [8,9] and *Trypanosoma brucei* [10] and viruses such as adenovirus [11] and astrovirus [12] from NHPs’ fecal samples. Most NHP species are listed under a category of conservation [13,14]. Molecular identification in fecal samples of blood-borne pathogens will be of great advantage to monitor NHP populations that can be a potential zoonotic reservoir for humans. 

Gastrointestinal parasites have been monitored in neotropical primates [5,15,16,17,18,19,20,21]; however, they are restricted to some countries such as Mexico [22,23,24,25,26,27,28,29,30,31,32,33] and Brazil [34,35,36,37,38,39,40,41,42,43,44]. Regarding the study of hemoparasites and arboviruses in neotropical primates, this one is restricted just to a few studies in Brazil [45,46,47,48], Venezuela [49,50] and French Guiana [49], and Ecuador has no data on them [51], even if most of those hemoparasites and arboviruses are present in Ecuador [52,53,54,55,56]. Finally, we wish to focus on hemoparasites and arboviruses because they are the cause of millions of infections and thousands of deaths per year in humans [56,57,58,59]. Understanding whether primates act as a wild reservoir for hemoparasites and viruses in the neotropical region will allow us to better understand the ecology of diseases [60] and the role of NHPs in the emergence of human diseases [61], as well as the way to implement control programs [62,63] for endemic [64] and incoming pathogens [65] and NHP conservation/management plans in Ecuador [66].

Some NHPs can become infected with hemoparasite species of protozoans. For example, wildlife harbors several species of *Plasmodium* [67,68,69], especially NHPs. However, in the neotropics, just recently, there is evidence of natural infection in humans with *Plasmodium brasilianum* in Venezuela [70] and *Plasmodium simium* in Brazil [45]. Therefore, in order to identify potential zoonotic reservoirs in wildlife, it is essential to monitor *Plasmodium* sp. in the Amazon region of Ecuador. Another example, *Toxoplasma* sp., has a worldwide distribution and affects a wide range of hosts from humans [71] and domestic animals [72] to wildlife [3,73], including marine mammals [74], freshwater mammals [75] and NHPs, Old World (OW) and New World (NW) monkeys [48,76,77,78,79]. In Ecuador, however, screening to detect *Toxoplasma* was only carried out in the islands of Galapagos. Indeed, some studies found *Toxoplasma* in birds [80,81], domestic animals [82], as well as in environmental waters. *Leishmania* sp. occurs in a wide range of hosts [83,84,85,86,87,88], including human [89] and non-human primates [48]. In NHPs, experimental [90] and natural infections [91] have been registered. However, studies in the neotropics are restrained to Brazil, and countries such as Ecuador are under-surveyed even though the parasite is widely distributed [92]. In Ecuador, despite surveillance and control campaigns, trypanosomiasis is still present [93]. However, there are just a few studies of trypanosomiasis in wildlife: bats [94,95], marsupials and rodents [96], and frogs [97], and unfortunately there are none on primates. In addition, in NW monkeys, it is very common to find several species of trypanosomes such as *Trypanosoma (megatrypanum) minasense* [98], and also zoonotic trypanosomes: *Trypanosoma rangeli* and *T. cruzi* [99]. 

Viral infections also pose a threat to NHPs’ health. Four types of viruses may affect NHPs: enveloped DNA (deoxyribonucleic acid) viruses, non-enveloped DNA viruses, enveloped RNA (ribonucleic acid) viruses and non-enveloped RNA viruses [100]. Among the latter, arboviruses (arthropod-borne viruses) are a diverse range of viruses from eight families: *Togaviridae* (genus *Alphavirus*), *Flaviviridae* (genus *Flavivirus*), *Peribunyaviridae* (example: genus *Orthobunyavirus*)*,* *Nairoviridae* (example: genus *Orthonairovirus*), *Phenuiviridae* (example: genus *Phlebovirus*), *Reoviridae* (genus *Orbivirus*), *Rhabdoviridae* (genus *Vesiculovirus*) and *Orthomyxoviridae* (genus *Thogotovirus*).

Arboviruses are a public health concern due to the threat to both humans and animals [101,102]. Arbovirus hosts can vary from a specific taxonomic group to several hosts. The range of vectors can also vary in the same way. For some arboviruses, the zoonotic origin is linked to primates because of their close genetic distance, while others are linked to other vertebrates or the vector itself [103]. In the neotropics, NHPs have been identified as hosts for the following diseases: yellow fever [104,105,106], Mayaro virus [49], Zika virus, Chikungunya virus [107], hepatitis A [108], Cacipacoré virus [109], St. Louis encephalitis virus (SLEV) and Oropouche virus (OROV) [49,110].

## 2. Results

### 2.1. Current Situation of Non-Human Primates

#### Non-Human Primate Biodiversity

Primates from all over the world are divided into two groups: Old World Monkeys (Catarrhinni) and New World Monkeys (Platyrrhini). Around the world, we reported 504 species, including 171 species in the Neotropical region (i.e., South America and Middle America). The Neotropical region is the zoogeographical region with the highest number of species, and Ecuador registers 21 species (Table 1). All groups are mainly arboreal and they play an important role in cultures [111], in religions [112], in human livelihoods [113], and in the threat of emerging diseases [105]. They are also a good indicator of the quality of the environment [114], and at this time the destruction of their habitats, hunting and the capture of live specimens for export and local use are the greatest threats to their conservation [115,116,117,118,119]. 

### 2.2. Terminology

#### 2.2.1. Key Concepts

Blood pathogens can infect NHPs. However, a lot of terms have been identified across studies. This is why we propose the following concepts based on international guidelines. A disease is considered to be an abnormal condition in one part of the body or in the entire animal with clinical signs [125]. An infectious disease is caused by an agent that infects a host and can be transmitted to other hosts [126]. Blood-borne pathogens are viruses, bacteria and parasites found in the blood that can cause a disease.

#### 2.2.2. Non-Invasive Samples and Detection Methods

The source of DNA in NHPs can be hairs [127,128], feces [129,130], buccal cells from swabs [131,132,133] or food wadges [134], urine [135] and blood [98,136]. Non-invasive genetic sampling was defined by Taberlet, et al. [137] as “*the source of the DNA left behind by the animal and that can be collected without having to catch or disturb the animal*”. Non-invasive samples have been used in several studies of a wide range of vertebrates, such as birds [138,139,140], marine mammals [141,142,143], wolves [144,145], amphibians [146,147], reptiles [148], fish [149,150] and non-human primates. Non-invasive samples are known to have low quality and low quantity of DNA [151,152,153]. Samples such as pure blood have better results, but their collection is considered to be invasive. There are even cases where wild animals have died when trapped or manipulated for sampling. Therefore, the use of non-invasive samples can minimize disturbance to animals when collected correctly. However, sometimes non-invasive samples can disturb the ecology of animals. For example, in animals where their feces is used to mark their territory [154], collecting the whole feces can disturb the territory of the animal. In conservation biology, the use of non-invasive samples is of the utmost importance when it comes to threatened or endangered species such as gorillas [155], and in some cases is legally mandated. 

Bacteria (*n* = 3), protozoa (*n* = 29) and viruses (*n* = 24) have been reported to infect the blood of neotropical NHPs (Table 2, Table 3 and Table 4). Studies in NHPs use invasive samples to detect blood pathogens. Most detection methods on protozoa focus on a combination between microscopy, polymerase chain reaction (PCR) and serological methods such as enzyme-linked immunosorbent assay (ELISA) (Table 3). However, for viruses, they focus primarily on a hemagglutination test (Table 4), which is considered a test for the presence of a humoral immune response of NHPs to an infectious agent such as viruses. 

### 2.3. Risk Factors Associated with the Transmission of Disease

There are several risk factors that favor the transmission of diseases such as socioecological and ecological factors (Table 5) [270,271].

#### 2.3.1. Socioecological Factors

- Animal behavior and social organization

Non-human primates are social, and as social animals they are at risk of infectious or parasitic diseases [272]. Factors such as group size, movement between groups, and sexual selection (number of mating partners) are among the variables of host–parasite interactions in NHPs that are considered to be drivers of parasite transmission [273]. Regarding group size, this variable is a risk factor for some infectious and parasitic diseases. In Amazonian primates, a larger group size will attract more mosquitos, and a higher risk of malaria infection was observed [274,275]. However, this is not true for other vectors [276]. There are strategies such as fission where subgrouping can act as a dilution effect for vectors [277]. The type of contact such as grooming [278] and the contact rate might also influence parasite transmission [279]. For example, lice transferred to other lemurs across several seasons [280] may increase the risk of infection of parasitic diseases [281,282]. Host age may also have an influence on the prevalence of hemoparasites in non-human primates [283]. For *Plasmodium*, the innate immune system plays a role in protecting young non-human primates from it and the parasite can benefit from an immune system weakened by age [284]. In Springer, et al. [285], *Plasmodium* sp. were more likely to infect older individuals of Verreaux’s sifakas (*Propithecus verreauxi*), whereas *Babesia* sp. infected the younger ones.

- Sleeping site ecology

Sleeping behavior has been described as a risk factor for parasite transmission. It has been suggested that Amazonian NHPs sleeping in microhabitats are less likely to be infected with malaria [275]. In Milne-Edwards’ sportive lemur (*Lepilemur edwardsi*), they are at greater risk of infection of ectoparasites and thus hemoparasites as well because they sleep in tree holes [286]. On the contrary, chimps (*Pan troglodytes schweinfurthii*) build their sleeping sites in *Cynometra alexandri* trees, which are known for having insect-repellent properties [287], decreasing the risk of infection.

- Migration

The OIE has not declared the presence of the West Nile virus in South America; however, three horses have died in Argentina and they were diagnosed with the WNV close to a North American cluster [288]. It has been suggested that wild bird migration could spread the virus to South America [289,290,291]. This scenario is not only valid for animals [292], but for humans as well [293]. The origins of *Plasmodium falciparum* and *Plasmodium vivax* in Central and South America are related to the migration of enslaved Africans and Australasian people, respectively [294].

#### 2.3.2. Ecological Factors

- Host density

Host density is another risk factor that can increase parasite transmission [295,296]. However, lower densities such as those from orangutans (*Pongo pygmaeus*) (around two individuals per km^2^) can harbor as many as two species of malaria [297].

- Climate change

Climate change influences the emergence of infectious and parasitic diseases in several types of environments [298,299]. Several studies have described potential scenarios with models of climate change to describe distribution patterns of hosts and their pathogens and/or the vector [300,301]. As for the impact of climate change on NHPs, some studies have described habitat shifts at the altitudinal gradient [302] or at the latitudinal gradient [303]. Nunn, et al. [304] published a study on the latitudinal gradient of parasite species richness, which can give us an idea of how this distribution can impact NHPs if these ones change their distribution. In the neotropics, according to the modeling of the IPCC [305], some forests will shift to savannah woodlands, where this process could influence the host–parasite interaction [306,307]. The density and diversity of pathogens might be different in these savannas than in the forest [308,309]. Climate change could lead to host switching in NHPs [310] and an increase in the distribution of vectors [311,312,313,314,315]. For example, in avian malarias, host specificity was found in regions with pronounced rainfall seasonality [316]. However, pathogens can also adapt to new temperatures [317]. Finally, climate change can indirectly influence the behavior of primates, which can influence how pathogens can spread across populations. For example, climate change has a direct influence on the phenology of plants (e.g., fruiting, flowering) [318], and as a consequence, groups of NHPs forage differently for food [319,320,321], whether they fission into subgroups or whether they increase their home range for more food [322]. The implications of these changes are reflected on the host and might change the host–parasite relationship. According to HobergandBrooks [323], the primary sources of emerging infectious diseases will be those that are going to be able to survive climate change.

#### 2.3.3. Human Activities

Human activities such as agricultural practices [324] and land-use changes (e.g., deforestation) [325,326] can increase the risk of parasite transmission [327]. For example, in Malaysian Borneo, macaque hosts and mosquito vectors are having more contact with humans due to these human activities [325]. In South America, there are also NHPs that survive in human-disturbed environments; thus, they can maintain the sylvatic cycle close to humans [69].

#### 2.3.4. Others

Malnutrition [328,329] due to poor habitats (e.g., fragmentation) [307] and toxic chemicals and pollution are other factors that also increase parasite transmission [328].

### 2.4. Surveillance Networks

#### 2.4.1. World Organization for Animal Health (OIE)

The animal health situation is monitored in each country and each country is responsible to declare to the OIE (World Organization for Animal Health). However, there are no notifications on NHPs for the Neotropical region or elsewhere. However, there are publications of some diseases such as yellow fever present in non-human primates [330]. For other diseases present in the OIE portal, the Ebola virus disease, for example, is not listed; however, they recommend it to be voluntarily reported [331,332]. In addition, the OIE have a guideline and a training manual on wildlife disease surveillance [333,334], which could be applied as guidelines in Ecuador and other neotropical countries.

#### 2.4.2. International Organizations from the United Nations System and Wildlife Monitoring

The World Health Organization (WHO) work in collaboration with the FAO (Food and Agriculture Organization of the United Nations) and OIE to deal with zoonotic diseases. However, they also work with local governments, academia as well as non-governmental organizations (NGOs). The FAO has several programs such as Vmergem, PAATS and LinkTads that have as objectives to help and to develop technical capacities for local governments. 

#### 2.4.3. Local Networks

The Ministry of Environment in Ecuador does not have a program on wildlife disease monitoring but it has workshops on wildlife health [335]. The National Institute of Research on Public Health (INSPI) has a program on parasites and infectious diseases, and they make guidelines for zoonotic wildlife diseases and wildlife groups such as NHPs. As for NGOs and management plans, there are none working on specific wildlife disease surveillance in Ecuador. However, the IUCN (International Union for Conservation of Nature) have international guidelines for each taxonomic group and their diseases, which can be applied by specialists all over the world [336,337,338]. Brazil is the only neotropical country with a guideline and a manual on epizootics in NHPs [339,340]. It is no coincidence that it is the country with the highest number of studies on neotropical NHP diseases (Table 2, Table 3 and Table 4).

## 3. Discussion

Diseases in NHPs are of conservation and medical importance because they may threaten both NHP populations [105,252] and humans [341,342]. That is why monitoring and long-term surveillance in NHPs [343,344] can enhance the knowledge of diseases and the risks associated with them. However, we should pay attention to the choice of methods to detect NHP diseases. For example, for neotropical NHPs, just one study used a non-invasive method to monitor protozoa [9] (Table 1, Table 2 and Table 3). Invasive techniques such as serological tests, blood smears, and tissues are used to detect arthropod-borne diseases and blood pathogen diseases [345]. Instead, you can use fecal [8,346,347], urine [348] or saliva [349,350] samples to monitor viruses, bacteria and other blood pathogens and obtain as much information as the other techniques as long as you only need to have an idea of the prevalence and the presence of the disease. Once you have a general idea of the current situation, in order to characterize the disease, you can move forward to an invasive technique but with fewer samples. 

It is important to use non-invasive samples in wildlife studies since there are studies that determined the diagnostic sensitivity of molecular tests for the study of blood-borne pathogens, and obtained data close to invasive samples [351]. For example, for *Plasmodium falciparum*, a study determined by PCR the limit of detection at 6.5 parasites/µL in fecal samples from NHPs from the Brazilian Amazon [9]. In human blood samples, the limit of detection of *Plasmodium falciparum* ranges from 0.03 parasites/µL to 9 parasites/ml using methods such as qPCR [352] and RT-PCR [353]. The sensitivity of parasite DNA extraction for both stool and blood samples will depend on sample storage [354], DNA extraction methods [355] and parasite densities in the population and in individuals [356,357]. Studies aim to improve molecular techniques to increase the sensitivity of these techniques in the diagnosis of pathogens [352,353,358,359].

Socioecological and ecological risk factors are associated with the transmission of blood-borne pathogens in NHPs. Factors such as human activities and climate change are identified as factors in the emergence of infectious diseases [360]. However, vectors must be considered to evaluate the transmission of these pathogens. For example, vector density and longevity would also increase the transmission rate of these pathogens [361]. Studies have even identified the feeding preferences of vectors and their connection to disease transmission [362,363]. Another study found an effect between habitat fragmentation and the infection rate of vectors with *Plasmodium* sp. [364].

Methods of surveillance should be adapted to wildlife populations. In captive settings, monitoring is easier than in wild populations. Additionally, the risk of infection can change whether they are captive or wild. Captive settings are an environment under control most of the time (depending on the captive conditions in neotropical countries), while monitoring free-ranging populations can be difficult for several reasons (poaching or legal hunting for meat, illegal pet trade, among others). However, long-term studies on NHPs may help to mitigate the effect of hunting [365]. NHPs from captive settings are most of the time from unknown origin [366], which makes it more difficult to know the biohazard threat involved. Sometimes the quarantine period is not respected, and diagnostic tests are not performed (either because they do not have the budget or because they are not aware of them), increasing the risk of infections. In addition to these conditions, the contact rate with humans such as care takers and tourists can introduce human pathogens to those populations (reverse zoonoses) [367], increasing the chances that an NHP can be infected. It is not unusual to see on social media, even during a pandemic, rescue center personnel or tourists taking pictures of themselves with primates without adequate biosecurity measures. In the other direction, pathogens can be transmitted to humans through primate biting (contact with body fluids) or scratches [368]. Cases of monkey bites in Ecuador are not unusual; however, local health services do not follow strict protocols such as taking samples from the patient and the monkey for further analysis or applying prophylaxis treatments against NHP bacteria or rabies. 

In order to reduce the risks associated with the diseases, local governments should implement control measures adapted to NHPs. There are high risk activities such as NHP translocations [369] (from one geographical region to another or from one captive setting to another), reintroductions [370], among others, that can be a health risk for local populations of NHPs and humans. The success of these high-risk activities depends not only on NHP health but also on NHP socioecology, the support from local communities and the presence of environmental education programs [371]. NHP local populations and translocated groups should be monitored constantly. The costs of these activities are really high and losing individuals would be a step backwards. If the risk is too high, maybe the budget associated with this activity should be implemented in other types of conservation programs that could help primate populations more than the same translocation or reintroduction.

## 4. Materials and Methods

**Figure 1:**
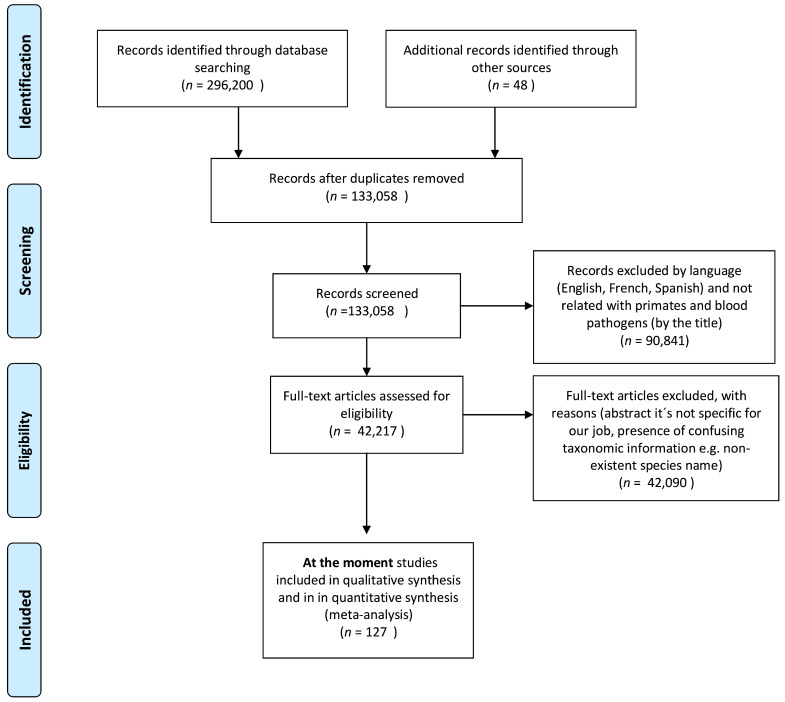
Flowchart (modified from PRISMA 2009) describing the literature search and study selection.

This systematic review was carried out using PRISMA guidelines for reporting systematic reviews and meta-analyses [372,373] and to identify bibliographic research from 1927 until 2019 about blood parasites, hemoparasites and arboviruses present in neotropical non-human primates. In several databases, we used the following search string (keywords and Boolean operators) “blood and parasites and primates”, “Hemoparasites and Primates”, “Haemoparasites and Primates”, “Arbovirus and Primates” or “Parasites and Primates”. The databases that we used were Scopus, Google Scholar and Pubmed. We also included grey literature such as theses and abstract presentations (Figure 1). Once the results were obtained, we made a selection by eliminating studies according to the following criteria: (1) the parasite was not a hemoparasite, (2) the published studies were in a language that the authors do not understand, (3) the study was not from a neotropical non-human primate, and finally (4) duplicate studies. We included all articles that clearly indicated the name of the parasite and the species of the host. We also included studies in captive and wild habitats.

## 5. Conclusions

In this study, we found that NHPs are reservoirs for a large number of blood-borne pathogens. In addition, socioecological and ecological risk factors facilitate the transmission of these blood-borne pathogens either between NHPs or between NHPs and humans. The genus *Alouatta* is the one that records the highest number of blood-borne pathogens. This genus has the widest range of distribution from Mexico to Argentina. However, bacterial and viral pathogen groups have not been studied in depth in South America and especially in Ecuador, so these data will allow decision makers to decide where to focus their research efforts.

The Ministries of Health and Environment should prioritize the implementation of infection prevention and control measures in countries with a high risk of disease transmission. The Ministry of Environment should have a protocol to protect workers who are exposed to zoonotic diseases, for example, park rangers and zoo care takers, but also ecotourism. Ecotourism is considered a vulnerable group but also a group that exposes NHPs to infections [374,375]. A guideline should establish measures to prevent the introduction and spread of infection among NHP and human populations [376]. Some measures include reducing the frequency and duration of field visits as well as the number of visitors. Another biosecurity measure is to increase the viewing distance to NHPs [125,377]. Additionally, we should consider surveillance in national programs [378] as a tool for public health [333] and NHP conservation [3,338,379,380]. Finally, there are a large number of diseases that are under-surveyed. A large number of studies support surveillance programs as they improve the early detection of diseases [381,382,383,384]. These surveillance programs must have regular and effective monitoring protocols adapted to non-human primates. In order to implement these control programs, Ministries of Environment, Universities, and Health and wildlife researchers must collaborate with each other to determine monitoring strategies and to identify priority diseases for the country.

## Figures and Tables

**Table 1:** Species of non-human primates from Ecuador.

Family	Primate Species	Size (cm)	Weight (g)	Group Size (# of Individuals)	Social System ^1^	Habitat ^2^
Aotidae	*Aotus lemurinus*	50	1300	Small (1 to 5)	M	TFF
	*Aotus vociferans*	50	698	Small (2–5)	M	TFF, FF, and crops
Atelidae	*Alouatta seniculus*	43–60	3600–9000	Small (3–7) or Large (15)	MM–MF or a group of bachelor males	FF
	*Alouatta palliata aequatorialis*	48–67	3100–9800	Small and Medium (2–12)	MM–MF	All types of forests
	*Ateles belzebuth*	40–60	6000–10,500	Large (≈20)	MM–MF	TFF
	*Ateles fusciceps fusciceps*	39–58	8800	Large (20–30)	MM–MF	TFF
	*Lagothrix lagotricha lagotricha*	40–58	3600–10,000	Large (6–60)	PG/MM–MF/MM	TFF
	*Lagothrix lagotricha poeppigii*	40–58	3600–10,000	Large (2–25)	PG/MM–MF/MM	TFF
Cebidae	*Cebus albifrons*	81–84	1900–3900	Medium (5–30)	PG/MM–MF	All types of forests
	*Cebus aequatorialis*	35–46	1200–3600	Medium (5–20)		
	*Sapajus macrocephalus*	35–50	1700–4500	Medium (5–20)		
	*Cebus capucinus capucinus*	35–45	2900–3900	Medium (2–42)		
	*Cebus yuracus*	81–84	2900–3900	Medium and Large (10 and 35–54)		
	*Saimiri cassiquiarensis macrodon*	25–32	600–1400	Large >10 and up to 100		
Callitrichidae	*Cebuella pygmaea*	12–15	100–140	Small and Medium (2–9)	M/PA	TFF, FF
	*Leontocebus nigricollis graellsi*	15–28	350–470	Small and Medium (2–30)	M/MM–MF	TFF and FF
	*Leontocebus lagonotus*	17–27	330–430	Medium (2–10)	All kinds of social structures	All kind of forest
	*Leontocebus tripartitus*	21–24	218–240	Small and Medium-sized (4–10)	PG	TFF and FF
Pithecidae	*Plecturocebus discolor*	28–36	900–1400	Small (2–5)	M	FF-lianas forest–forest gaps
	*Cheracebus lucifer*	30–38	800–1500	Small (2–5)		TFF
	*Pithecia aequatorialis*	39–44	2000–2500	Small (1–4)		TFF
	*Pithecia milleri*	37–48	2100–2600	Small (2–6)		TFF and FF
	*Pithecia napensis*	37–48	2100–2600	Small and Medium (4–8)		TFF

^1^ Social system: M = Monogamous: A mating system in which only one male copulates with only one female; MM–MF = Multimale–Multifemale groups: A social group consisting of multiple adult males and multiple adult females; MM = Multi-males: Strong social relationships among males, often deriving from close kinship among those males as a result of male philopatry; PA = Polyandry: A mating system in which one female copulates with multiple males; PG = Polygynandry: A mating system in which members of both sexes copulate with multiple members of the opposite sex [120]; ^2^ Habitat: TFF—Terra firme forest: forest with soils composed of clay or loam [121]; FF—Flooded forests: Forests characterized by trees waterlogged or submerged during a large part of the year [122]. Data on NHPs were obtained from the following references: [123,124].

**Table 2:** Bacteria found in neotropical non-human primates.

Bacteria	Host	Location	Sampling (Invasive Non-invasive)	Detection Methods ^1^	References
*Borrelia burgdorferi*	*Leontopithecus chrysomelas*	Brazil	Invasive	Nested PCR	[156]
*Leptospira* spp.	*Ateles fusciceps* *Ateles geoffroyi vellerosus * *Ateles geoffroyi yucatanensis * *Ateles hybridus* *Callithrix jacchus* *Callithrix pennicilata* *Cebus* sp. *Cebus albifrons* *Cebus capucinus* *Leontopithecus* sp. *Leontopithecus chrysomelas* *Saguinus leucopus* *Saimiri sciureus* *Sapajus apella*	Brazil Colombia French Guiana Mexico	Invasive	PCR MAT Serology	[157] [158] [159] [160] [161] [162] [163] [164] [165]
*Mycoplasma* spp.	*Alouatta* sp. *Alouatta caraya* *Saimiri sciureus* *Sapajus apella* *Sapajus flavius* *Sapajus nigritus* *Saguinus midas niger*	Brazil French Guiana	Invasive	TEM PCR	[166] [167] [168] [169] [170] [171] [172]

^1^ TEM = Transmission electron microscopy; MAT = Modified agglutination test.

**Table 3:** Protozoa found in neotropical non-human primates.

Protozoa	Host	Location	Sampling (Invasive Non-Invasive)	Detection Methods	References
*Babesia* sp.	*Alouatta seniculus* *Ateles paniscus* *Callithrix* sp. *Pithecia pithecia* *Saimiri sciureus*	French Guiana	Invasive	BS	[173] [68]
*Leishmania* sp. *Leishmania (Viannia)*	*Alouatta guariba* Atelidae (unknown species) *Aoutus azarai azarai* *Aotus nigriceps* *Callicebus nigrifrons* *Callithrix jacchus* *Callithrix penicillata* *Cebus macrocephalus* *Lagothrix cana* *Leontopithecus crysomelas* *Pithecia* sp. *Pithecia irrorata* *Saguinus imperator* *Saimiri ustus madeirae* *Sapajus apella* *Sapajus xanthosternos*	Argentina Brazil	Invasive	ELISA PCR IIF DAT PCR-RFLP	[174] [175] [91] [176] [177]
*Leishmania amazonensis*	*Ateles paniscus*	Brazil	Invasive	DNA PCR and RFLP	[178]
*Leishmania braziliensis*	*Saguinus geoffroyi*	Panama	Invasive	NA ^1^	[179]
*Leishmania chagasi* *Leishmania infantum*	*Callicebus nigrifrons* *Callithrix jacchus*	Brazil	Invasive	PCR IHC DAT	[91] [180]
*Leishmania mexicana*	*Alouatta palliata* *Alouatta pigra*	Mexico	Invasive	ELISA IIF Western Blot	[181]
*Leishmania (Viannia) shawi*	*Chiropotes satanus* *Sapajus apella*	Brazil	Invasive	Monoclonal antibodies	[85]
*Plasmodium* sp.	*Alouatta seniculus*	Brazil	Invasive	Conventional microscopy (GIEMSA) PCR	[182]
*Plasmodium vivax*	*Alouatta caraya* *Alouatta guariba clamitans* *Alouatta seniculus macconnelli* *Sapajus apella*	Brazil French Guiana	Invasive	Microscopy IFA ELISA PCR Real-time PCR	[183] [184] [185] [186] [187]
*Plasmodium brasilianum*	*Alouatta* sp. *Alouatta seniculus* *Alouatta seniculus straminea* *Alouatta caraya* *Alouatta guariba clamitans* *Alouatta guariba guariba* *Aotus nigriceps* *Ateles* sp. *Ateles belzebuth* *Ateles chamek* *Ateles paniscus* *Aotus nigriceps* *Brachytheles arachnoides* *Cacajao calvus* *Cacajao rubicundus* *Callicebus bruneus* *Callicebus dubuis* *Callicebus moloch* *Callicebus personatus* *Callicebus torquatus* *Callithrix geoffroyi* *Cebus* sp. *Chiropotes albinasus* *Chiropotes chiropotes* *Chiropotus* sp. *Chiropotes satanas* *Lagothrix cana cana* *Lagothrix lagotricha lagotricha* *Lagothrix lagotricha poeppigii* *Leontopithecus chrysomelas* *Leontopithecus rosalia* *Mico humeralifer* *Pithecia monachus* *Pithecia irrorata* *Pithecia pithecia* *Saguinus martinsi martinsi* *Saguinus martinsi ochraceous* *Saguinus midas niger* *Saguinus midas* *Saimiri* sp. *Saimiri sciureus* *Saimiri sciureus sciureus* *Saimiri sciureus boliviensis* *Saimiri ustus* *Sapajus apella apella* *Sapajus apella macrocephalus* *Sapajus robustus* *Sapajus xanthosternos*	French Guiana Brazil Venezuela	Invasive	BS Conventional microscopy (GIEMSA) PCR ELISA	[188] [68] [189] [182] [190] [191] [192]
*Plasmodium simium*	*Alouatta guariba clamitans* *Callicebus nigrifrons* *Cebus* sp. *Sapajus robustus* *Sapajus xanthosternos*	Brazil	Invasive Non-Invasive	BS PCR PCR from fecal samples Nested PCR	[193] [174] [183] [190] [9]
*Plasmodium falciparum*	*Alouatta caraya* *Alouatta guariba* *Alouatta puruensis* *Alouatta seniculus macconnelli* *Ateles chamek* *Callicebus bruneus* *Lagothrix cana cana* *Sapajus apella*	Brazil French Guiana	Invasive	ELISA IFA PCR	[188] [185]
*Toxoplasma* sp.	*Alouatta seniculus*		Invasive	DAT	[9]
*Toxoplasma gondii*	*Alouatta* sp. *Alouatta belzebul* *Alouatta caraya* *Alouatta seniculus* *Alouatta seniculus straminea* *Ateles* sp. *Ateles geoffroyi* *Ateles paniscus paniscus* *Aotus* sp. *Aotus nigriceps* *Brachyteles arachnoides* *Cebus* spp. *Cebus albifrons* *Cebus capucinus* *Callithrix* sp. *Callithrix penicillata* *Chiropotes satanas* *Erythrocebus* sp. *Leontopithecus* sp. *Leontopithecus chrysomelas* *Leontopithecus chrysopygus* *Leontopithecus rosalia* *Leontopithecus rosalia rosalia* *Lagothrix lagotricha* *Saguinus imperator* *Saguinus labiatus* *Saguinus oedipus* *Saimiri* sp. *Saimiri boliviensis* *Saimiri sciureus* *Saimiri sciureus sciureus* *Sapajus* spp. *Sapajus apella* *Sapajus libidinosus* *Sapajus flavius* *Pithecia pithecia pithecia*	Argentina Brazil Colombia French Guiana Mexico	Invasive Necropsy	DAT IFAT MAT PCR Latex agglutination kit Indirect hemagglutination kit ELISA IHC PCR-RFLP Histology	[194] [195] [196] [197] [198] [199] [200] [201] [202] [203] [204] [205] [206] [207] [48] [208] [209] [210] [211] [212] [213] [214] [215] [76] [216] [217] [218]
*Trypanosoma* sp.	*Alouatta seniculus* *Ateles paniscus* *Pithecia pithecia* *Saguinus leucopus* *Saimiri sciureus*	Colombia French Guiana Panama	Invasive	BS	[68] [219] [220]
*Trypanosoma cruzi*	*Alouatta palliata* *Alouatta pigra* *Alouatta caraya* *Alouatta seniculus* *Ateles belzebuth* *Ateles geoffroyi* *Ateles fusciceps* *Aotus* sp. *Aotus azarai* *Aotus nigriceps* *Cacajao calvus* *Callicebus personatus* *Callicebus nigrifrons* *Callithrix geoffroyi* * Callithrix jacchus* *Callithrix penicillata* *Cebuella pygmaea* *Cebus albifrons* *Cebus capucinus* *Cheracebus torquatus* *Chiropotes satanas* *Leontopithecus chrysopygus* *Leontopithecus chrysomelas * *Leontopithecus rosalia* *Leontocebus fuscicollis* *Leontocebus fuscicollis weddelli* *Leontocebus nigricollis* *Mico chrysoleucus* *Mico argentatus* *Mico emiliae* *Pithecia irrorata* *Plecturocebus brunneus* *Saguinus niger* *Saguinus geoffroyi* *Saguinus bicolor bicolor* *Saguinus imperator imperator* *Saguinus labiatus* *Saguinus leucopus* *Saguinus midas* *Saguinus mystax* *Saguinus ustus* *Saimiri boliviensis* *Saimiri sciureus* *Saimiri ustus* *Sapajus libidinosus* *Sapajus robustus* *Sapajus xanthosternos*	Argentina Brazil Mexico French Guiana Panama Peru	Invasive	ELISA IIF PCR BS IFA HC XD	[181] [221] [203] [222] [223] [179] [224] [225] [226] [227] [228] [229] [230] [231]
*Trypanosoma devei*	*Cebuella pygmaea* *Callimico goeldii* *Leontocebus fuscicollis weddelli* *Leontocebus tamarin tamarin* *Saguinus imperator imperator*	Brazil	Invasive	HC	[231] [232] [233]
*Trypanosoma diasi*	*Sapajus apella apella*	Brazil	Invasive	HC	[232]
*Trypanosoma forestali*	*Alouatta guariba* *Alouatta caraya*	Argentina Brazil	NA ^1^	NA ^1^	[223]
*Trypanosoma hippicum*	*Alouatta guariba* *Alouatta seniculus*	NA	NA ^1^	NA ^1^	[219]
*Trypanosoma lambrechti*	*Alouatta seniculus* *Cebus albifrons* *Cheracebus torquatus* *Chiropotes satanas* *Pithecia pithecia* *Sapajus apella*	Brazil	NA ^1^	NA ^1^	[219] [179] [223]
*Trypanosoma lesourdi*	*Ateles paniscus*	French Guiana	NA ^1^	NA ^1^	[223]
*Trypanosoma mycetae*	*Alouatta belzebul* *Alouatta belzebul belzebul* *Alouatta palliata* *Alouatta caraya* *Alouatta seniculus* *Chiropotes satanas*	Brazil Guatemala Panama French Guiana	Invasive	XD Direct observation of blood	[229] [233] [232] [223] [224] [219]
*Trypanosoma minasense*	*Alouatta belzebul* *Alouatta caraya* *Alouatta guariba* *Alouatta seniculus* *Aotus trivirgatus* *Ateles fusciceps* *Ateles geoffroyi griscescens Callithrix jacchus* *Callithrix penicillata* *Cebus albifrons* *Cebus capucinus* *Leontocebus weddelli* *Leontocebus fuscicollis weddelli* *Plecturocebus ornatus* *Saguinus geoffroyi* *Saguinus imperator imperator* *Saguinus midas* *Saimiri sciureus* *Saimiri sciureus macrodon* *Saimiri ustus* *Sapajus apella*	Argentina Brazil Colombia French Guiana Panama Peru	Invasive	PCR Stained films of peripheral blood	[232] [179] [203] [229] [234] [223] [98] [224] [231] [230] [235]
*Trypanosoma rangeli (like)*	*Alouatta seniculus* *Cebuella pygmaea* *Cebus albifrons unicolor* *Cebus capucinus* *Callimico goeldii* *Leontocebus fuscicollis weddelli* *Pithecia pithecia* *Saguinus bicolor* *Saimiri boliviensis* *Saimiri ustus* *Saimiri sciureus* *Saguinus geoffroyi* *Saguinus imperator imperator* *Saguinus midas* *Saimiri boliviensis* *Sapajus apella*	Brazil Colombia French Guiana Panama Peru	Invasive	BS	[236] [203] [237] [224] [230] [231] [229]
*Trypanosoma saimiri*	*Saimiri sciureus sciureus*	Brazil	Invasive	HC	[233]
*Trypanosoma venezuelensis*	*Alouatta guariba* *Alouatta seniculus*	NA ^2^	NA ^2^	NA ^2^	[219]

^1^ BS = Blood smears; ELISA = Enzyme-Linked Immunosorbent Assay; IIF = Indirect Immunofluorescence Assay; DAT = Direct Agglutination Test; MAT = Modified Agglutination Test; IFAT = Indirect Fluorescent Antibody Technique; IFA = Immunofluorescence Assay; IHC = Immunohistochemistry; XD = Hemoculture Xenodiagnosis; HC = Hemoculture. ^2^ N.A.: Not available.

**Table 4:** Viruses found in neotropical non-human primates.

Virus	Host	Location	Sampling (Invasive Non-Invasive)	Detection Methods ^1^	References
Arbovirus (not specified)	*Alouatta caraya* *Sapajus* sp. *Sapajus apella*	Brazil	Invasive	HA	[238] [109]
Eastern equine encephalitis virus	*Ateles paniscus chamek* *Sapajus libidinosus*	Bolivia Brazil	Invasive	Antibody titers (IgG and IgM antibodies) HI	[239] [240]
Western equine encephalitis virus	*Cebus libidinosus*	Brazil	Invasive	HI	[240]
Alphavirus (not specified)	*Sapajus apella*	Brazil	Invasive	HA	[238]
Mayaro virus	*Alouatta villosa* *Alouatta seniculus* *Callithrix argentata* *Pithecia pithecia* *Saguinus midas* *Sapajus apella* *Sapajus libidinosus*	Brazil Panama French Guiana	Invasive	HA Serologic (PRN antibodies) HI	[238] [203] [241] [240] [242] [243]
Una virus	*Alouatta caraya*	Argentina Paraguay	Invasive	NTAb survey	[244]
Venezuelan equine encephalitis virus	*Sapajus apella*	Colombia	Invasive	NTAb survey	[245]
Mucambo virus	*Sapajus libidinosus*	Brazil	Invasive	HI	[240]
Flavivirus (not specified)	*Leontopithecus chrysomelas* *Sapajus apella* *Sapajus xanthosternos*	Brazil	Invasive	HA HI	[238] [246]
West Nile virus	*Alouatta caraya*	Argentina	Invasive	RT-nested PCR	[247]
Yellow fever virus	*Alouatta* sp. *Alouatta caraya* *Alouatta clamitans* *Alouatta guariba clamitans* *Alouatta fusca* *Alouatta macconnellii* *Alouatta seniculus* *Ateles paniscus chamek* *Callicebus* sp. *Callithrix* sp. *Cebus* sp. *Leontopithecus* sp. *Leontopithecus chrysomelas* *Pithecia pithecia* *Saguinus midas* *Sapajus* sp. *Sapajus libidinosus*	Argentina Bolivia Brazil Colombia French Guiana Panama Trinidad Venezuela	Invasive Necropsy	Immunoperoxidase staining method HI ELISA IgM / inoculated in mice (Isolation by RT-PCR) IHC RT-qPCR RT-PCR Serologic (PRN antibodies) Antibody titers (IgG and IgM antibodies) Isolation in cell cultures (*Aedes albopictus* C3/36), followed by DFA, RT-PCR generic for Flavivirus, and genome sequencing IFAT IIF using monoclonal antibodies	[248] [249] [250] [251] [246] [252] [203] [105] [239] [240] [253] [254] [255] [256] [257] [258] [242] [259] [260]
Ilheus virus	*Alouatta caraya* *Callithrix jacchus* *Callithrix penicillata* *Leontopithecus chrysomelas* *Sapajus libidinosus*	Argentina Brazil	Invasive	HI NT RT-nested PCR	[261] [246] [240] [247] [262]
Saint Louis encephalitis virus	*Alouatta caraya* *Alouatta seniculus* *Ateles paniscus chamek* *Leontopithecus chrysomelas* *Pithecia pithecia* *Saguinus midas* *Sapajus nigritus* *Sapajus cay* *Sapajus libidinosus*	Brazil Argentina French Guiana	Invasive	HI NT MNT RT-nested PCR	[261] [246] [240] [239] [203] [247] [263]
Rocio virus	*Leontopithecus chrysomelas* *Sapajus libidinosus* *Sapajus xanthosternos*	Brazil	Invasive	HI	[246] [240]
Zika virus	*Callithrix* sp. *Leontopithecus chrysomelas* *Sapajus* sp.	Brazil	Invasive	HI	[246] [264]
Dengue virus	*Alouatta caraya*	Argentina	Invasive	RT-nested PCR	[247]
Bussuquara virus	*Alouatta caraya* *Leontopithecus chrysomelas* *Leontopithecus chrysopigus* *Saguinus bicolor*	Argentina Brazil	Invasive	RT-nested PCR NT HI	[246] [247] [265]
Cacicapore virus	*Leontopithecus chrysomelas*	Brazil	Invasive	HI	[246]
Orthobunyavirus	*Leontopithecus chrysomelas* *Sapajus apella*	Brazil	Invasive	HI	[246] [266]
Oropouche orthobunyavirus	*Alouatta caraya* *Callithrix* sp. *Sapajus apella* *Sapajus libidinosus*	Brazil	Invasive	HA HI Neutralization assays CF confirmed by RT-PCR	[238] [267] [240] [268]
Apeu virus	*Alouatta caraya* *Sapajus apella*	Brazil	Invasive	PRN T70	[269]
Tacaiuma orthobunyavirus	*Leontopithecus chrysomelas*	Brazil	Invasive	HI Real time PCR	[246]
Phlebovirus	*Leontopithecus chrysomelas*	Brazil	Invasive	HI	[246]
Icoaraci phlebovirus	*Alouatta caraya* *Leontopithecus chrysomelas*	Brazil	Invasive	HI NT	[246,261]

^1^ HA = Hemagglutination test; HI = Hemagglutination inhibition test; NATb = Neutralizing antibody (NTAb) survey; BS = Blood smears; ELISA = Enzyme-Linked Immunosorbent Assay; IHC = Immunohistochemistry; PRN = Plaque-reduction neutralizing; DFA = Direct Immunofluorescence Assay; IFAT = Indirect Fluorescent Antibody Technique; IIF = Indirect Immunofluorescence Assay; NT = Neutralization test; MNT = Mouse neutralization test; CF = Complement fixation test.

**Table 5:** Risk factors associated with the transmission of diseases in neotropical non-human primates.

Risk Factors	Factors	Examples
Socioecological factors	Animal behavior and social organization	Group size Movement between groups Sexual selection number of mating partners Type of contact and contact rate Host age
	Sleeping site ecology	Habitat
	Migration	Animal Humans
Ecological factors	Host density	High vs. low
	Climate change	Habitat shifts Host switching Primate behavior
	Human activities	Agricultural practices Land-use changes
	Others	Malnutrition Pollution

## Data Availability

The data that support the findings of this study are available from the corresponding author upon reasonable request.
